# The Dynamics of *Deformed Wing Virus* Concentration and Host Defensive Gene Expression after *Varroa* Mite Parasitism in Honey Bees, *Apis mellifera*

**DOI:** 10.3390/insects10010016

**Published:** 2019-01-08

**Authors:** Yazhou Zhao, Matthew Heerman, Wenjun Peng, Jay D. Evans, Robyn Rose, Gloria DeGrandi-Hoffman, Michael Simone-Finstrom, Jianghong Li, Zhiguo Li, Steven C. Cook, Songkun Su, Cristina Rodríguez-García, Olubukola Banmeke, Michele Hamilton, Yanping Chen

**Affiliations:** 1 USDA-ARS Bee research Laboratory, Bldg. 306, BARC-East, Beltsville, MD 20705, USA; zhaoyazhou@caas.cn (Y.Z.); Matthew.Heerman@ARS.USDA.GOV (M.H.); Jay.evans@ars.usda.gov (J.D.E.); leejh6972@126.com (J.L.); zhiguo.li@fafu.edu.cn (Z.L.); Steven.Cook@ARS.USDA.GOV (S.C.C.); cristinarodriguez.crg@gmail.com (C.R.-G.); olubukola.banmeke@bison.howard.edu (O.B.); Michele.hamilton@ars.usda.gov (M.H.); 2 Institute of Apicultural Research, Chinese Academy of Agricultural Sciences, Beijing 100093, China; pengwenjun@vip.sina.com; 3 USDA APHIS, National Program Manager for Honey Bee Health, Riverdale, MD 20737, USA; Robyn.I.Rose@aphis.usda.gov; 4 USDA-ARS Carl Hayden Bee Research Center, 2000 East Allen Road, Tucson, AZ 85719, USA; Gloria.Hoffman@ARS.USDA.GOV; 5 Honey Bee Breeding, Genetics and Physiology Research Laboratory, USDA-ARS, Baton Rouge, LA 70820, USA; Michael.SimoneFinstrom@ars.usda.gov; 6 College of Bee Science, Fujian Agriculture and Forestry University, Fuzhou 350002, China; susongkun@zju.edu.cn; 7 Laboratorio de Patología Apícola, Centro de Investigación Apícola y Agroambiental, IRIAF, Consejería de Agricultura de la Junta de Comunidades de Castilla-La Mancha, 19180 Marchamalo, Spain

**Keywords:** honeybee, *Varroa*-DWV interaction, DWV titers, immune gene expression, anti-pathogen mechanisms

## Abstract

The synergistic interactions between the ectoparasitic mite *Varroa destructor* and *Deformed wing virus* (DWV) lead to the reduction in lifespan of the European honey bee *Apis mellifera* and often have been implicated in colony losses worldwide. However, to date, the underlying processes and mechanisms that form the multipartite interaction between the bee, mite, and virus have not been fully explained. To gain a better understanding of honey bees’ defense response to *Varroa* mite infestation and DWV infection, the DWV titers and transcription profiles of genes originating from RNAi, immunity, wound response, and homeostatic signaling pathways were monitored over a period of eight days. With respect to DWV, we observed low viral titers at early timepoints that coincided with high levels of Toll pathway transcription factor Dorsal, and its downstream immune effector molecules *Hymenoptaecin*, *Apidaecin*, *Abaecin*, and *Defensin 1*. However, we observed a striking increase in viral titers beginning after two days that coincided with a decrease in Dorsal levels and its corresponding immune effector molecules, and the small ubiquitin-like modifier (SUMO) ligase repressor of Dorsal, PIAS3. We observed a similar expression pattern for genes expressing transcripts for the RNA interference (Dicer/Argonaute), wound/homeostatic (Janus Kinase), and tissue growth (Map kinase/Wnt) pathways. Our results demonstrate that on a whole, honey bees are able to mount an immediate, albeit, temporally limited, immune and homeostatic response to *Varroa* and DWV infections, after which downregulation of these pathways leaves the bee vulnerable to expansive viral replication. The critical insights into the defense response upon *Varroa* and DWV challenges generated in this study may serve as a solid base for future research on the development of effective and efficient disease management strategies in honey bees.

## 1. Introduction

The European honeybee (*Apis mellifera*) is the predominantly managed pollinator and provides economically important pollination services worldwide that are estimated to exceed $153 billion annually [1,2]. However, the past decade, beekeepers, in the United States and Europe, have been facing annual hive losses of over 30 percent or higher raising serious public and societal concerns [3,4,5]. According to recent studies, pathogens, pesticides, climate change, landscape alteration, agricultural intensification and their cumulative interactions have contributed extensively to honey bee colony losses [6,7,8,9,10,11].

Of critical importance is the question of how all of these deleterious facets that affect survival and performance of honey bee colonies are correlated to the ectoparasitic mite *Varroa destructor*; a primary agent of colony decline [7,12]. *Varroa* mite infestations occurred originally only in the Asian honey bee *Apis cerana*, and was first introduced to the European honey bee over 70 years ago [13]. Over the course of the 20th century, *Varroa’s* host range has spread to nearly every beekeeping region of the world [13,14]. *Varroa* feed on the hemolymph of pupal and adult stages of honey bees, not only weakening the bees and shortening their lifespan but also vectoring viruses and other pathogens [13].

*Deformed wing virus* (DWV) is a positive stranded RNA virus belonging to the family *Iflaviridae* and has been the subject of intensive investigation due to its widespread infection in honey bee colonies and close association with *Varroa* mite infestations [15]. In the absence of *Varroa*, DWV is maintained at low and often asymptomatic levels in the colony through vertical transmission, [16,17], horizontal transmission, and trophallaxis. However, *Varroa* is an efficient vector and activator of DWV because virus particles are injected directly into bee hemolymph during mite feeding and virus replication is stimulated through host immunosuppression [18,19,20], although one study noted a correlation between increased DWV and immune pathway responses after wounding bees and placing them in non-sterile cells to finish development [21]. These feeding behaviors by *Varroa* allow DWV to be transmitted horizontally within the bee colony and between bee colonies. DWV impairs pupal development andshortens lifespans of adult bees [21]. With respect to prevalence, *Varroa* may increase the percentage of infected bees in a colony from 10 to 100%, and drive DWV concentration 1 million fold higher [22]. High levels of DWV replication allow for enriched in-colony genetic diversity, which ultimately gives way for more virulent forms of the virus. The association of *Varroa* mites DWV has killed billions of honey bees across the globe over the past 50 years [22].

The underlying mechanisms that drive the switch from covert or latent towards incipient or actively replicating infections in bees parasitized by *Varroa* have been studied in some detail. *Varroa* may have some capacity for suppressing bee antiviral mechanisms [19] and drive selection for more virulent strains [23]. Immune responses, including Toll signal pathway that induces antimicrobial peptide (AMP) gene expression, and their connected homeostatic and cell growth pathways are associated with controlling the persistence of pathogen infection in bees [24]. While these pathways engage in cross-talk at various levels, they certainly maintain distinct hierarchies and should be considered, in part, as an individual phenomenon [25]. Wnt signaling pathway is an important regulator of development and immune function including Toll signaling pathway [26,27]. The protein Naked cuticle (nkd) is a negative regulator of the Wnt signaling pathway and limits the effects of the Wnt signaling. Activation of Toll pathway immunity is conserved from insects to mammals and occurs via the expression of NF-κB (*Dorsal-1A* in honey bees), and the synthesis of antimicrobial peptides (AMPs) [24]. The link between mite feeding and honey bee immune dysfunction is via *Dorsal-1A* expression during *Varroa* feeding [28]. Using the AMP *Apidaecin* as a readout, it was observed that reduction in *Dorsal* and *Apidaecin* coincided with hemolymph feeding by *Varroa*. Additionally, it was observed that increased DWV concentrations resulted in reduced encapsulation and melanization within bees [20].

RNA interference (RNAi) via double stranded RNA (dsRNA) is a sequence specific post-transcriptional gene-regulatory and the principal insect innate immune response for the detecting and inhibiting virus replication in insects. The constant evolutionary arms race has a profound effect on the outcome of host-virus interactions [29]. There are many kinds of RNA viruses that infect different developmental stages of honey bees. The role of RNAi in mediating dsRNA-induced antiviral response in honey bees was confirmed in several studies. Depending on the level of immune response elicited, manifestations of viral infections are varied ranging from completely asymptomatic to producing overt deformities, paralysis, or death [24,30].

In our study, we probed transcriptional profiles originating from multiple pathways involved in immunity, homeostasis, to determine the effects of *Varroa* parasitism and DWV infection. We demonstrate a *Dorsal* dependent expression pattern that coincides with DWV concentrations AMP expression, homeostasis, cell metabolism, and proliferation pathways.

## 2. Materials and Methods

### 2.1. Honey Bees Sample Collection

Honeybees were collected from apiaries of *Apis mellifera ligustica*, which were managed by the USDA-ARS Bee Research Laboratory, Beltsville, MD, USA. All treatment of honeybees conformed with the laws of the USA in relation to the care and use of laboratory animals. To reduce the influence of physiological and genetic variation, all freshly emerged workers were obtained from brood frames originating from the same colony. This colony was confirmed free of *Varroa* infestation and had no detectable pathogens based on our monthly survey for parasites and pathogens following the methods described previously [31]. Frames with sealed brood were removed from the colony, placed in a mesh-walled cage individually and transferred to an insect growth chamber at 34 °C and 70% humidity. After a 48 hour incubation, newly-emerged bees were collected for subsequent experimental setup.

### 2.2. *Varroa* Mite Collection

*Varroa* mites were collected from four bee colonies infested with mites. The colonies were not treated with miticides and were kept in an isolated apiary of the USDA-ARS Bee Research Laboratory in Beltsville, MD. The positive status of DWV infection of the bee colonies was confirmed by sampling bees and *Varroa* mites from each hive and performing an RT-PCR assay following the previously-described method [32]. *Varroa* mites from the colonies were collected using a Powdered Sugar Roll method [33]. Briefly, brood frames covered with adult workers were individually removed from bee colonies and about 200–300 worker bees were shaken into a wide-mouthed Mason jar filled with three tablespoons of powdered sugar and sealed with 1/8 inch (0.3175 cm) wire mesh lid. After gently rolling the jar a few times to ensure that all of the bees were coated with powdered sugar and then waiting for 1–2 min, mites were dislodged from the bees by turning jar upside down and shaking vigorously for one minute over a white-colored plastic container half-filled with water. Mites floating on the surface of the water were collected by pouring the water through a fine mesh cloth and transferred to a petri dish with a piece of filter paper placed on the bottom. The bees were returned to the colony.

### 2.3. *Varroa* Challenging Experiment

Our previous studies demonstrated that *Varroa* mites can serve as a vector to facilitate the transmission of viruses between bees. Bees that were exposed to 30% *Varroa* mites will all became virus- positive under laboratory conditions [33,34]. As a result, a 30% level of *Varroa* infestation was set for our challenge experiment to monitor the dynamics of DWV concentrations and gene expression in honey bee hosts. Forty emerging honey bees were collected and placed in bee rearing cups [35]. A feeder made of 3-mL Luer-Lok syringe and filled with 1:1 ratio of honey and water solution was inverted over the top of the rearing cup to provision the caged bees. Bees were also provided with 1.5 g of pollen patty (BeePro©, Mann Lake, Hackensack, MN, USA), supplemented 10% (*w*/*w*) with ground fresh pollen, and placed on onto the bottom of the rearing cup. Twelve *Varroa* mites representing an infestation of 30% were introduced into each bee rearing cup. Twelve rearing cups (12 × 40 = 480 bees) were established with *Varroa* and three rearing cups (3 × 40 = 120 bees) without mites served as the negative control. Seven bees were collected from each rearing cup at five time points (0.5 day, one day, two days, four days, and eight days) post the introduction of *Varroa* mites. Two mites were also collected at the each time point post treatment to maintain the 30% *Varroa* infestation level throughout the experiment. All collected bees and mites were stored immediately at −80 °C for subsequent RNA isolation and molecular analysis.

### 2.4. RNA Extraction and qRT-PCR

Total RNA was extracted from individual bees using TRIzol regent (Thermo Fisher Scientific, Inc., Waltham, MA, USA) following the manufacturer specifications. The obtained RNA pellet was dissolved in DNase/RNase-free water containing ribonuclease inhibitor (Thermo Fisher Scientific, Inc. ). RNA concentration and purity were evaluated using a Nano-Drop 8000 spectrophotometer (Thermo Fisher Scientific, Inc.). All RNAs were stored at −80 °C for later use.

Quantitative real-time reverse transcriptase-PCR (qRT-PCR) was carried out using a CFX384 Touch real-time PCR system (Bio-Rad, Hercules, CA, USA) with SYBR green as the fluorophore and *β-actin* as a reference gene. The primer information for qRT-PCR is described in Table 1. In order to normalize the quantitation result of each target, qRT-PCR was also performed for an internal control *A. mellifera* β-actin for each sample under the same amplification conditions. Of multiple housekeeping genes evaluated in our pilot study, β-actin has been proven the most effective one in qPCR data normalization. The approximately equal amplifying efficiencies of β-actin and DWV, as well as honey bee genes involving host defensive were confirmed in our pilot studies. Total volume of the qRT-PCR reaction mixtures was 12.5 μL consisting of 6.25 μL of 2× Brilliant^®^ SYBR green qRT-PCR 1-step Master Mix (Agilent, Santa Clara, CA, USA), 0.375 μL of each 20 μM forward and reverse primers, 0.5 μL of RT/RNase block enzyme mixture, 0.5 μL of RNA, and 4.5 μL of RNase free ddH_2_O. The PCR amplification program was as follows: 50 °C for 30 min, 95 °C for 10 min, followed by 40 cycles of 95 °C for 30 s, 59 °C or 55 °C (Table 1) for 60 s, and 72 °C for 60 s, followed with a final extension at 72 °C for 10 min. Dissociation melt-curves were also included at the end of each run for quality and specificity control. Each RNA sample was run in triplicate.

### 2.5. Data Analysis

The relative amounts of DWV and expression of genes originating from RNAi, immunity, wound response, and homeostatic signaling pathways between the *Varroa*-challenged group and Negative control groups at each time point were calculated with the 2^−ΔΔCt^ method [38]. The amplification result for each target was expressed as the threshold cycle (CT) value, which represents the number of cycles needed to generate fluorescent signal above a predefined threshold. The mean value and standard deviations of each target was normalized using the Ct value (the number of cycles needed to generate a fluorescent signal above a predefined threshold) corresponding to the β-actin following the formula: ΔCt = (Average Ct target) − (Average Ct β-actin). The group that had the lowest value was chosen as a calibrator and power transformed to 1. The ΔCt value of each group was subtracted by ΔCt value of the calibrator to yield ΔΔCt. Concentrations of DWV and honey bee transcripts between the treatment and negative control groups relative to the concentration of β–actin in honey bees were determined by 2^−ΔΔCt^ and expressed as fold-change [38].

After confirmation of a normal distribution and an equal variance of data, the unpaired Student’s *t*-test was used to determine if there was a significant difference (*p* ≤ 0.05) between the treatment group and negative control for each target at each time point post treatment. All the figures were generated using Graphpad Prism 7 (GraphPad Software, Inc., San Diego, CA, USA).

## 3. Results

### 3.1. Rapid Increase in DWV Concentration Coincided with Initial Increase Followed by Subsequent Downregulation of Dorsal and Concomitant AMP

**Figure 1:**
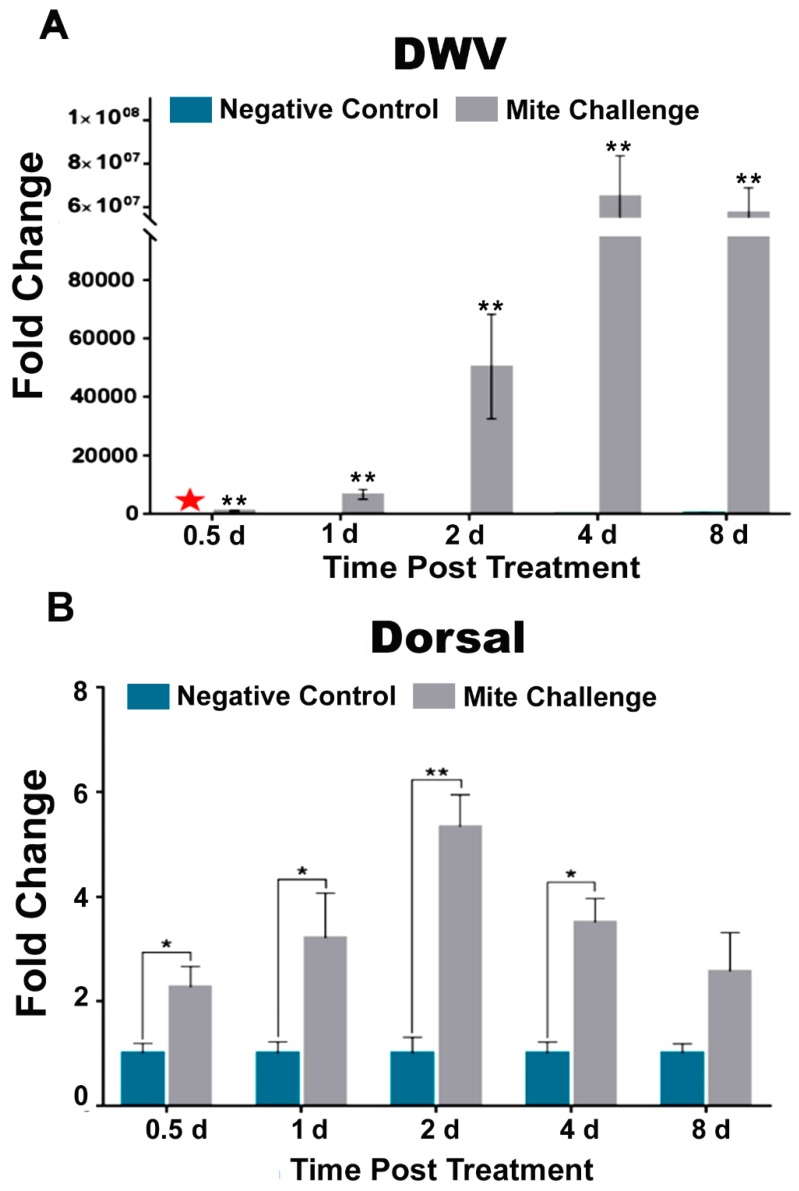
The concentration of DWV and the expression levels of the transcription factor Dorsal. (**A**) Fold changes of DWV between the negative control (without *Varroa* challenge) and the groups challenged with *Varroa* mites at the different time points post treatment. Bees infested with *Varroa* mites show significantly higher levels of the DWV compared to bees without exposed to *Varroa* challenge (star denotes calibrator; ** *p* < 0.01). (**B**) Fold changes between the negative control (cyan) and challenged bees (grey) at the different time points post treatment (* *p* < 0.05, ** *p* < 0.01).

**Figure 2:**
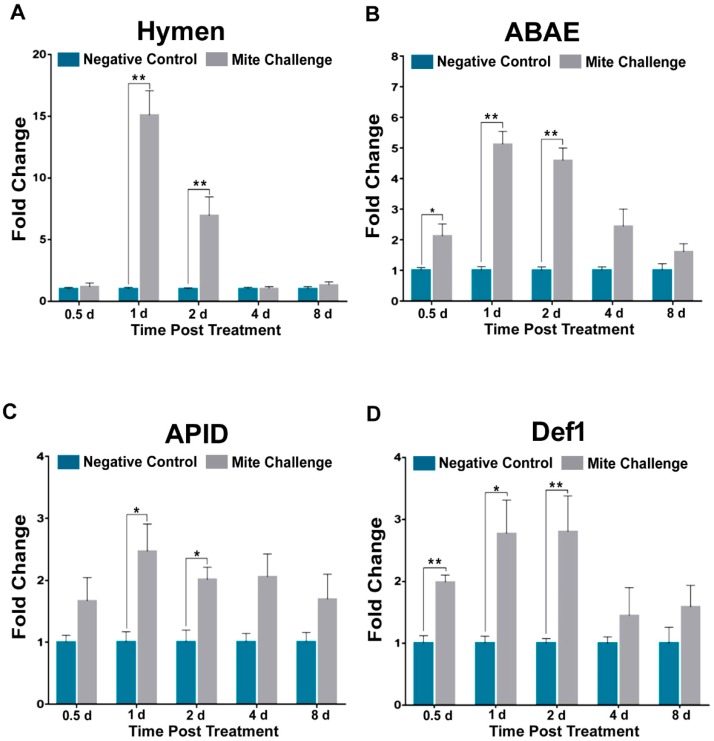
Fold change of the transcription profiles for Toll pathway AMPs downstream of *Dorsal* signaling between the negative control (without *Varroa* challenge) (cyan) and the bees challenged with *Varroa* mites (grey) at different time points post treatment (days). (**A**) Hymenoptaecin (Hymen), (**B**) Abaecin (ABAE), (**C**) Apidaecin (APID), and (**D**) Defensin 1 (Def1) showing similar transcription profiles for their activator Dorsal. (* *p* < 0.05, ** *p* < 0.01).

At every time point, DWV levels were significantly higher (*p* < 0.01) in bees exposed to *Varroa* (Figure 1A). More specifically, viral levels increased rapidly after 0.5-day post treatment, and reached their maximal levels at four days (Figure 1A). *Dorsal*, a transcription factor associated with the active expression of immune effector molecules, has been previously shown to be linked to high *Varroa* infestation and DWV concentrations [20]. Here we observed that Dorsal transcript levels significantly (*p* < 0.05) increased at approximately two-fold 0.5 days post treatment and peaked (*p* < 0.01) at nearly six-fold two days post treatment (Figure 1B). After which, levels began to drop and DWV levels rose (Figure 1). The aforementioned study [20] employed *Abaecin* as a readout for Toll immune response in *Varroa* infested bees. Here we probed *Hymenoptaecin* (Hymen), *Abaecin* (ABAE), *Apidaecin* (APID), and *Defensin 1* (Def1) as output relative to the down-regulation of Toll immunity. We observed that Hymen and ABAE transcriptional levels were elevated between 15-fold (*p* < 0.01; former) and five-fold (*p* < 0.01; latter) within the same window of DWV concentration increase and peak *Dorsal* expression (1-2d post treatment Figure 1A,B). A similar pattern was observed one and two days post treatment for two more AMPs, APID (*p* < 0.05) and Def1 (*p* < 0.01 and *p* < 0.05 respectively) (Figure 2C,D). The noted increases in expression at day 1 and day 2 post challenge were followed by decreases to levels similar to those of the uninfested bees by day 4 for each of the AMPs (Figure 2) and day 8 for *Dorsal* (Figure 1B) as DWV levels reached to peak (Figure 1A).

### 3.2. RNAi Pathway Transcript Levels Peak at Approximately the Same Time Post Challenge as Dorsal and AMPs

**Figure 3:**
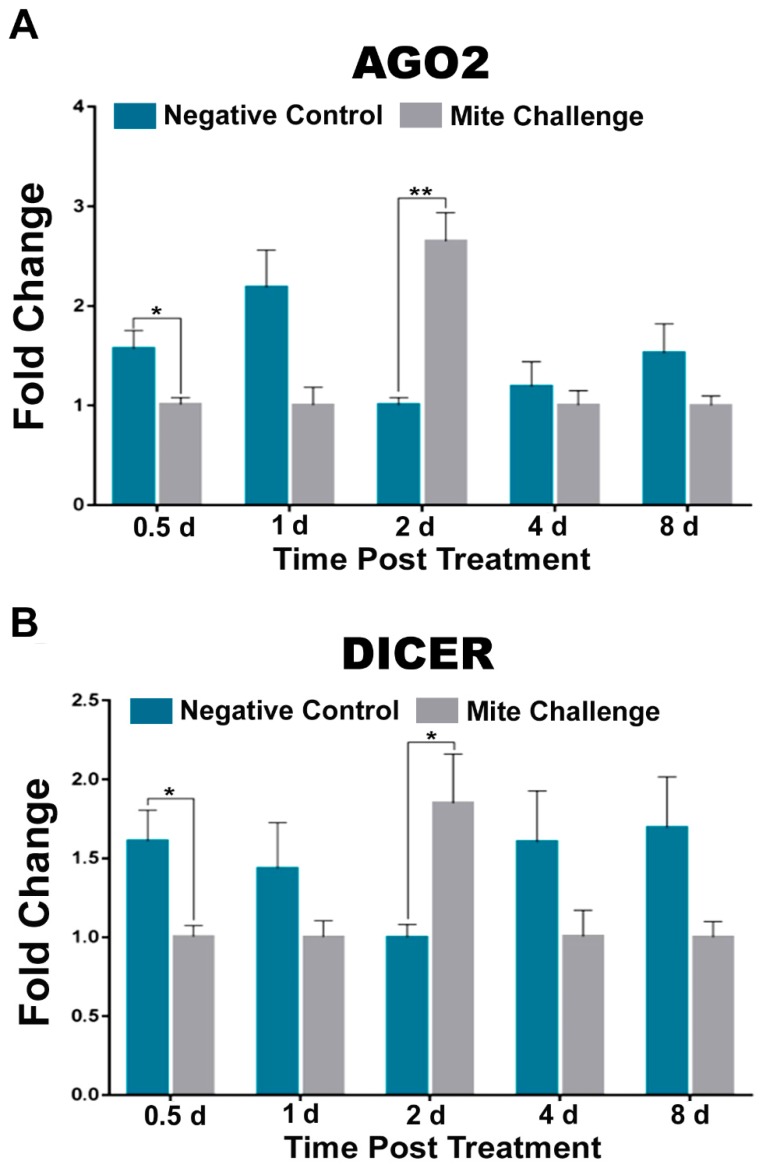
Fold change of the transcription profiles for components of RNAi pathway between the negative control (without *Varroa* challenge) and bees challenged with *Varroa* mites at different time points post treatment (days). (**A**) dsRNA binding AGO2 and (**B**) ribonuclease Dicer transcripts demonstrating down-regulation at all timepoints, except for significant upregulation two days post treatment. (* *p* < 0.05, ** *p* < 0.01).

The use of dsRNA molecules to disrupt translation of specific genes (RNAi) is a common molecular method to probe function. However, in animals, specifically insects [29], this phenomenon likely serves as a mode of antiviral defense. When we probed two components of the RNAi response in *A. mellifera*, we observed that at two days post treatment argonaute 2 (AGO2) and the ribonuclease (DICER) were both significantly upregulated (*p* < 0.01 and *p* < 0.05, respectively) in challenged bees. At every other time point, unchallenged bees had higher levels of these transcripts (Figure 3A,B).

### 3.3. TOR, Jak-STAT, and EGFR Pathways Mimic, to Varying Degrees, Dorsal Transcription in the Presence of *Varroa* Infestation

**Figure 4:**
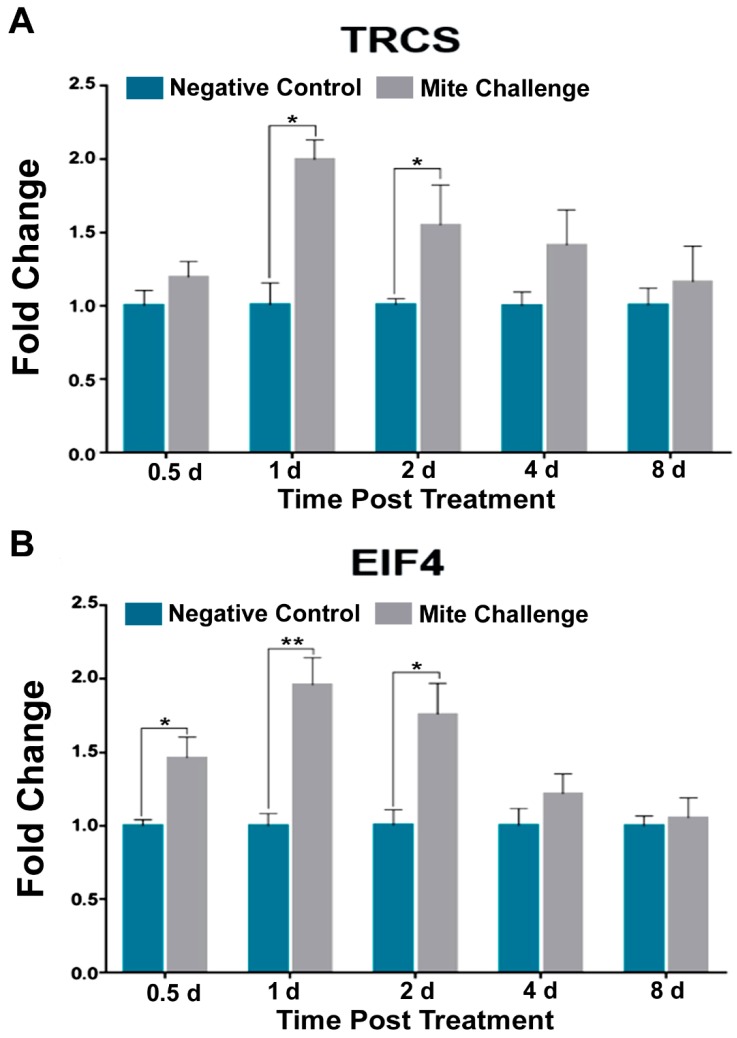
Fold chance of the transcription profiles for pieces of TOR pathway between the negative control (without *Varroa* challenge) and bees challenged with *Varroa* mites at different time points post treatment (days). (**A**) TOR subunit TRCS and (**B**) translation initiation factor EIF4 demonstrating similar expression patterns to Toll pathway components of immunity (* *p* < 0.05, ** *p* < 0.01).

Target of TOR is typically associated with nutrition and development. After *Varroa* infestation, we observed two-fold increases at one day post treatment for both the TOR subunit (TRCS; *p* < 0.01) and the eukaryotic translation initiation factor (EIF4); *p* < 0.05), and approximately 1.5-fold increases at two days (*p* < 0.05) for both transcripts (Figure 4A,B) in challenged bees.

**Figure 5:**
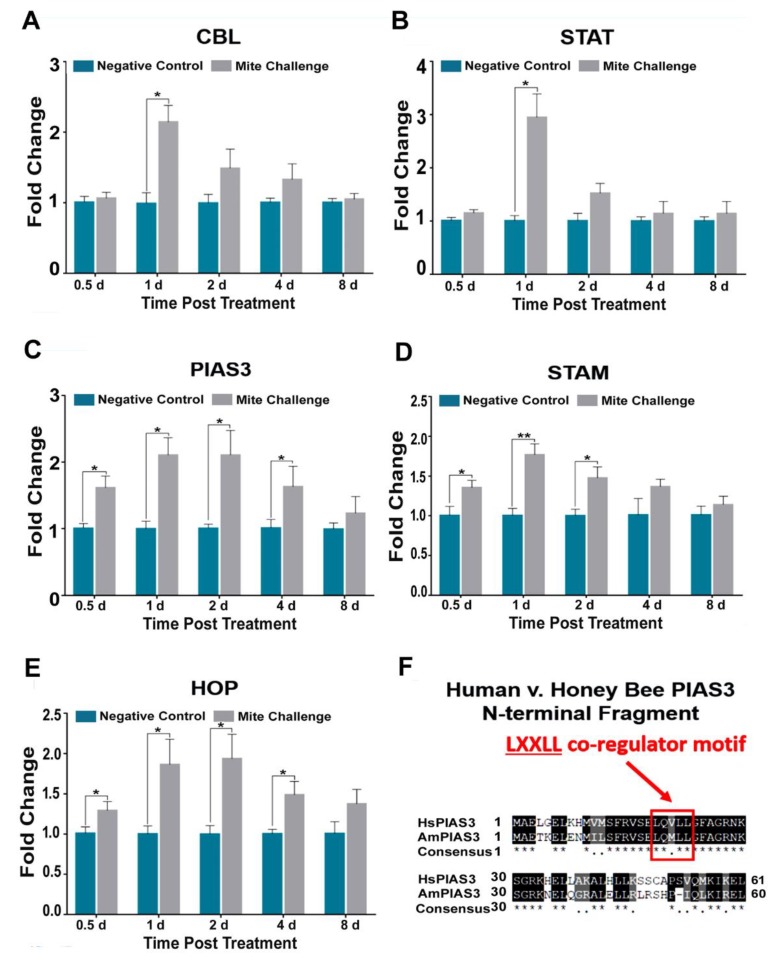
Fold chance of the transcription profiles for components of JAK-STAT pathway between the negative control (without *Varroa* challenge) and bees challenged with *Varroa* mites at different time points post treatment (days). (**A**) E3-ubiquitin ligase CBL, (**B**) transcription initiation factor STAT, (**C**) SUMO-ligase PIAS3, (**D**) signal adapter STAM, and (**E**) tyrosine kinase HOP demonstrating similar expression patterns to Toll pathway components of immunity (* *p* < 0.05, ** *p* < 0.01). (**F**) Multiple sequence alignment of the first 60 amino acids of human (HsPIAS3) versus honey bee (AmPIAS3). The LXXLL co-regulator motif found in humans is conserved in honey bees with a single neutral change from valine to methionine (Red).

The Jak-Stat signaling pathway has an important role in the control of immune responses. We observed that E3-ubiquitin ligase (CBL) and the transcription factor STAT levels were elevated nearly two and three-fold after one day of treatment, respectively, for challenged bees (*p* < 0.04; Figure 5A,B). SUMO-ligase (PIAS3), adapter (STAM), and tyrosine hopscotch (HOP) all followed transcriptional profiles that were similar to *Dorsal* levels for challenged bees. We observed a near two-fold increase for PIAS3 (*p* < 0.05; Figure 5C), STAM (*p* < 0.01 and *p* < 0.05; Figure 5D), and HOP (*p* < 0.05; Figure 5F). In humans, the E3 SUMO ligase PIAS3 acts as a negative regulator of NF-κB signaling. Here we demonstrate via multiple sequence alignment that the N-terminal co-regulator sequence motif that is minimally required for NF-κB repression in humans [39] is conserved in honey bees (highlighted in red; Figure 5F).

**Figure 6:**
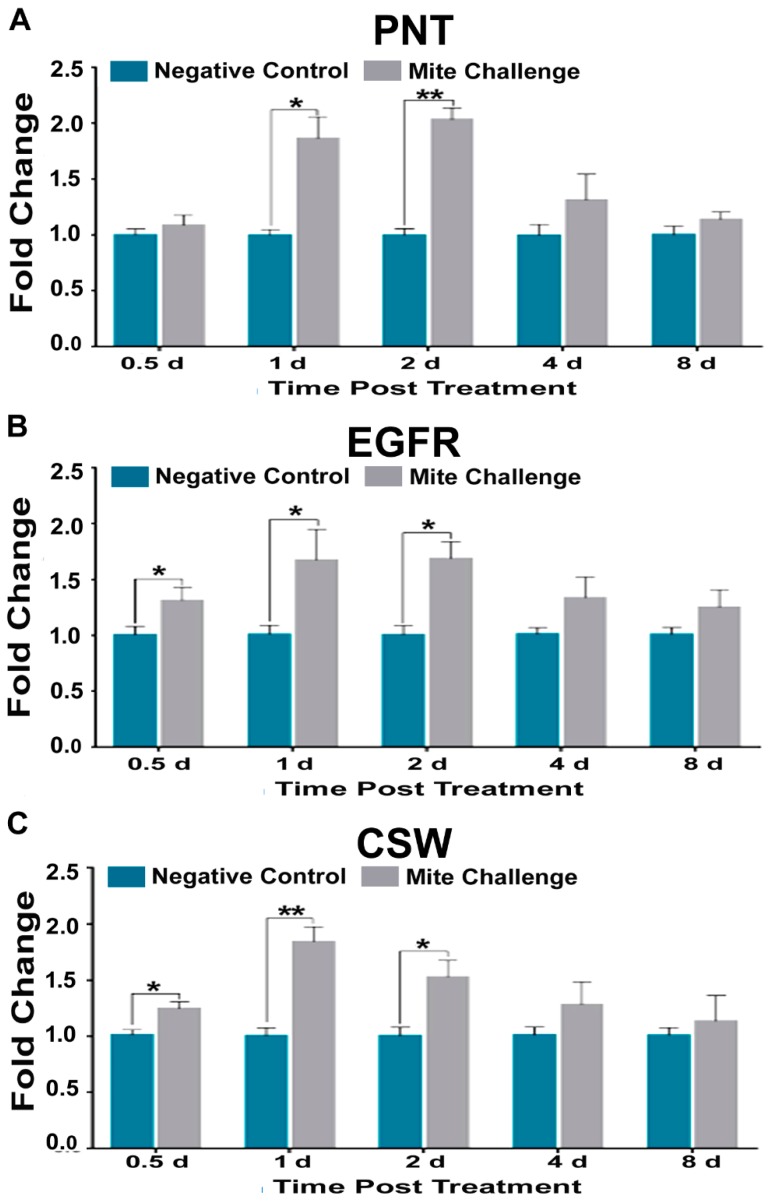
Fold chance of the transcription profiles for parts of cellular proliferation and growth pathway (EGFR) between the negative control (without *Varroa* challenge) and bees challenged with *Varroa* mites at different time points post treatment (days). (**A**) growth factor pointed (PNT), (**B**) receptor (EGFR), (**C**) and phosphatase corkscrew (CSW), demonstrating similar expression patterns to Toll pathway components of immunity (* *p* < 0.05, ** *p* < 0.01).

Our observations included a two-fold increase for the growth factor pointed (PNT) one day (*p* < 0.05) and two days (*p* < 0.01) post treatment (Figure 6A). Additionally, we found that the growth factor receptor (EGFR) had a similar increase in expression occurring between 0.5–2 days post treatment (*p* < 0.05; Figure 6B). The protein phosphatase corkscrew (CSW) also showed increases that peaked two-fold at one day (*p* < 0.01), approximately 1.5-fold at two days (*p* < 0.05), and returned to unchallenged levels at later time points.

### 3.4. Groucho, Protein Phosphatase 2b, and Rho1 Upregulation in Late Infections Correspond to High DWV Concentrations

**Figure 7:**
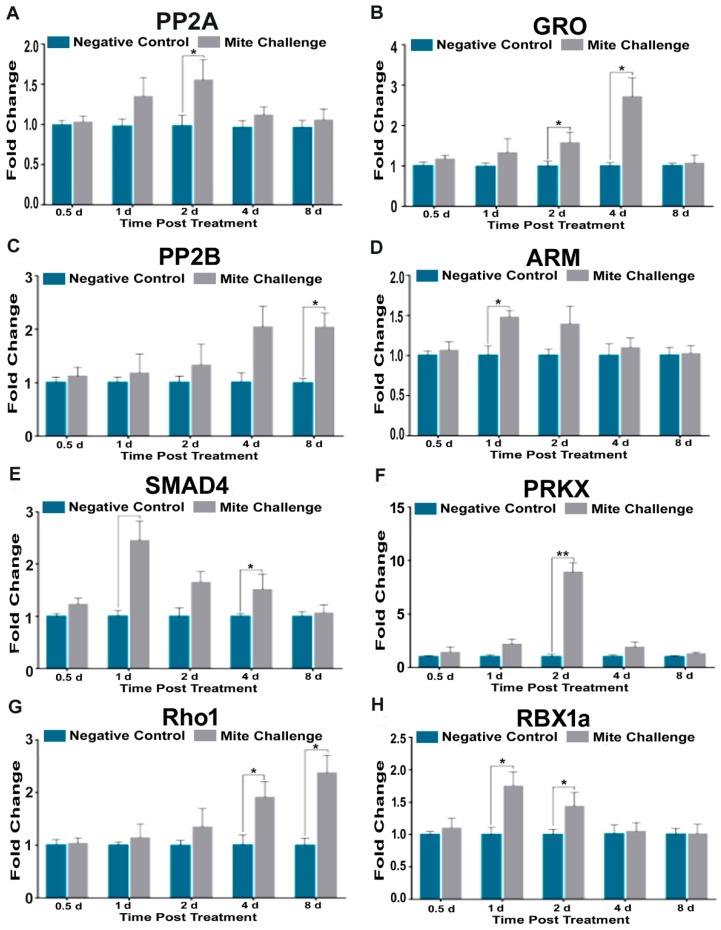
Fold chance of the transcription profiles for multiple elements of Wnt signaling between the negative control (without *Varroa* challenge) and bees challenged with *Varroa* mites at different time points post treatment (days). (**A**) phosphatase 2A (PP2A), (**B**) transcriptional repressor groucho (GRO), (**C**) phosphatase 2B (PP2B), (**D**) segment polarity gene armadillo (ARM), (**E**) transcription factor SMAD4, (**F**) kinase PRKX, (**G**) GTP binding protein Rho1, and (**H**) E3-ubiquitin ligase RBX1a demonstrating similar expression patterns to Toll pathway components of immunity (* *p* < 0.05, ** *p* < 0.01).

We assayed eight transcriptional profiles for genes originating from the Wnt signaling pathway. *Protein phosphatase 2A* (PP2A, Figure 7A), segment polarity gene *Armadillo* (ARM, Figure 7D), transcription factor (SMAD4, Figure 7E), kinase (PRKX, Figure 7F), and E3-ubiquitin ligase (RBX1a, Figure 7H) all followed the transcriptional profiles described for previous pathways. However, the Wnt transcriptional repressor *groucho* (GRO), peaked at a three-fold increase four days post *Varroa*-challenge with mites (*p* < 0.05; Figure 7B). *Protein phosphatase 2B*, and GTP-binding *Rho1* reached their approximately two-fold maximal transcriptional levels 8d after mite challenge (Figure 7C,G). These results coincide with the time points where *Varroa* infestation results in near maximal DWV levels (Figure 1A).

## 4. Discussion

There are numerous studies on *Varroa* parasitism and virus transmission and replication in honey bees [19,20,31,40]. These studies encompass various experimental designs, bee developmental stages, tissues, routes of infections, and time points post infestation and infection. We focused our efforts on a single laboratory method and infestation level and deeply probed the transcriptional profiles of multiple separate (yet invariably coupled) physiological pathways with respect to *Varroa* mites and DWV. Our attempts were to observe novel transcriptional irregularities and broaden the scope of what is known, by possibly identifying more players in the complex and multipartite interactions among bees, mites, and pathogens.

Of rather keen importance is the comparison of our methodology to other rigorous and validated studies. The importance of the Toll pathway in immunity has been shown not only in relation to *Varroa* mite infestation and DWV [20,28], but pesticides and the other bee viruses, as well [30,41]. While our study does not address pesticide concerns, our results with respect to the increase in DWV levels (Figure 1A) after mite infestation, and its coincidence with *Dorsal* transcription (Figure 1B) are comparable to previous studies [20]. Additionally, the aforementioned study [20] used *Apidaecin* as a readout marker for Toll activation, whereas we used AMPs *Hymenoptaecin*, *Apidaecin*, and *Defensin* 1 as in our previous study with *N. cerenae* [42]. Another recent study demonstrated no difference in *Hymenoptaecin* levels after infestation [43]. These results highlight the degree of accuracy and precision that can be expected from our experimental design and provide the basis for making a more comprehensive investigation of honey bee pathways when faced with high levels of mite feeding. This allowed us to observe a specific pattern of expression in RNAi, homeostatic, cell proliferation, and wound responses that closely aligned with the expression of *Dorsal*. While we observed this overarching pattern, in the spirit of brevity our remarks focus on genes not well explained or that provide novel targets for investigation.

As previously mentioned, *Dorsal* (NF-κB) suppression is a major contributor to immune dysfunction, and generally thought to be a genetic phenomenon unique to in honey bees [20]. However, this view does not take into account that the activity of the protein Dorsal is heavily influenced by the proteolysis of its repressors. When we measured the Jak-STAT pathway for PIAS3, a SUMO-ligase, we observed a two-fold increase in expression two days post challenge and beyond (Figure 5C). PIAS3 is crucial for the suppression of NF-κB signaling in mammals [37,44]. In *Drosophila*, the role of PIAS (ZIMP) has been demonstrated during development. This result is the first to implicate PIAS3 in honey bee immunity. Due to its sequence specific similarity to human PIAS3 (Figure 5F), this suggests that there may be a post-translational level of regulation of *Dorsal* in honey bees that has not been previously addressed. Downstream of Jak-STAT cytokine production is EGFR signaling. Here we observed that transcriptional levels for honeybee EGFR increased significantly beginning at 0.5 days post *Varroa* challenge up to two-fold at two days post challenge (Figure 6B). With respect to the RNA virus Hepatitis C, EGFR plays a crucial host gene role for virus infectivity [45]. 

Functionally, RNAi has been used to suppress viral levels within honey bees [30,46,47,48,49]. However, its endogenous role in the suppression or enhancement of viral infection has not been explored in bees as it has in fruit flies [50] and mosquitos [51,52]. Our observation that dsRNA binding protein AGO2 and ribonuclease DICER are upregulated during the crucial two days post-mite challenge period suggest an intrinsically important role in this pathway during that period that may contribute to the conversion of a latent (covert) DWV infection into an incipient and active (overt) infection. 

RNA viruses typically are translated without the aid of the canonical 5’ capping mechanism. EIF4, a translation initiation factor that occurs in both plants and animals supplants this need in multiple RNA virus infections. In plants, the Barley yellow dwarf relies on the action of EIF4 for translation [53], as well as the human viruses Coxsackievirus [54] and Foot and Mouth virus [55]. Within insects EIF4 has been associated only with RNA Rhabdovirus and then in the negative sense of RNA Rhabdovirus [56]. Interestingly the role of EIF4 in honey bee RNA viruses has yet to be elucidated. Here we observed a modest, albeit significant increase in EIF4 at two days post mite challenge when we probed the TOR pathway (Figure 4B).

Groucho (GRO) is a known transcriptional repressor of Wnt signaling expression in animals. While usually associated with insect development, in honey bees it has been recently implicated that is also influenced by the interactive effects of *Varroa* infestation and availability of pollen [57]. Here we provide evidence that this transcript may be acting to repress Wnt signaling in *Varroa* infested bees susceptible to high DWV infection levels (Figure 7B). This finding is the first to associate GRO expression with RNA virus activity. In mammals, GRO functions to promote the growth of glioblastomas [58]. PP2B and Rho1 are two other genes we found upregulated at later time points where high DWV levels were observed, and neither of these genes have been observed to be directly involved in infection and replication of viruses in animals. Notwithstanding, this does not eliminate them as possible targets for further investigation.

## 5. Conclusions

While it is understood that *Varroa* infestation causes immunosuppression concomitant to an increase in DWV concentrations in honey bees, we provides a transcriptionally-based global and temporal view of how the deleterious effects of *Varroa* feeding are involved with multiple pathways that are intimately linked to insect immunity. We provide evidence that there is disruption of key players involved with wound response, homeostasis, and cellular regeneration pathways in *Varroa*-induced viral replication.

## Figures and Tables

**Table 1:** Primers used for qRT-PCR in this study.

Gene Name	Abbr.	Accession No.	Primers (5′-3′)	T_m_ (°C)	Pathway	Reference
*Actin related protein 1*	*β-actin*	NM_001185145	F: TTGTATGCCAACACTGTCCTTT	—	Reference	[36]
			R: TGGCGCGATGATCTTAATTT			
*Deformed wing virus*	*DWV*	NC_004830.2	F: CGAAACCAACTTCTGAGGAA	55	Viral	[37]
			R: GTGTTGATCCCTGAGGCTTA			
*Signal transducer and activator of transcription 5B*	*STAT*	XM_397181	F: ATCAGTGGCTTCCAGCTACG	59	Jak-STAT	This study
			R: ATCGAAGGCAGCTCAGGATG			
*E3 SUMO-protein ligase PIAS3*	*PIAS3*	XM_623568	F: ACCACCTGCTCATCAAGCAT	59	Jak-STAT	This study
			R: AGCTGCACTTGTTGTTTGTTGT			
*Signal transducing adapter molecule 1*	*STAM*	XM_623536	F: GTCAGCCAGTACCGGAACAA	59	Jak-STAT	This study
			R: AGGTTGTTGGCTTACCGGAG			
*Tyrosine-protein kinase hopscotch*	*HOP*	XM_001121783	F: TTGTGCTCCTGAAAATGCTG	59	Jak-STAT	This study
			R: AACCTCCAAATCGCTCTGTG			
*E3 ubiquitin-protein ligase CBL*	*CBL*	XM_016911574	F: CCGGACACCGAGGAAATCAT	59	Jak-STAT	This study
			R: CGGTGGTGGAGCATTCTCTT			
*ETS-like protein pointed*	*PNT*	XM_016914525	F: TATACCGCGTTAGTGCTGCC	59	MAPK	This study
			R: CTAGCATCCCGCTTGATGGA			
*Epidermal growth factor receptor-like*	*EGFR*	XM_006560026	F: GTGAACAGTGCGAAGACGAA	59	MAPK	This study
			R: GGAACAATACGGTTCGCTGT			
*Tyrosine-protein phosphatase corkscrew*	*CSW*	XM_006561366	F: TTGCTGCTTCTCTTGCTTCA	59	MAPK	This study
			R: GTTCTGCTTGCATTCGTTGA			
*Target of rapamycin complex subunit lst8*	*TRCS*	XM_393223	F: TGTGGATGGCACTAACAGCA	59	TOR	This study
			R: ACCCTCTTCCTGAAAACCCA			
*Eukaryotic translation initiation factor 4 E type 3-A-like*	*EIF4*	XM_392604	F: TGGTGCTTGCAGCTATTGGA	59	TOR	This study
			R: GGCATGATGTGATTGATGAGGTTT			
*Embryonic polarity protein*	*Dorsal*	XM_016913118	F: CTCATCGGAAGACATGACAGTGA	59	NF-kB	This study
			R: TGAATTCAAAGCCAGTTCGAAAA			
*Protein argonaute-2*	*AGO2*	XM_395048	F: ACCTGCTGAGTTATGCACAGT	59	RNAi	This study
			R: AGCCTTTAGAACTCTTGCTGGT			
*Endoribonuclease Dicer*	*Dicer*	XM_016917734	F: AGCAGTAGCTGATTGTGTGGA	59	RNAi	This study
			R: TGAAGGATGTGTAAACGCCTGT			
*Hymenoptaecin*	*HYMEN*	NM_001011615	F: CTCTTCTGTGCCGTTGCATA	59	Toll	This study
			R: GCGTCTCCTGTCATTCCATT			
*Abaecin*	*ABAE*	NM_001011617	F: ATCTTCGCACTACTCGCCAC	59	Toll	This study
			R: AGCCTTGAGGCCATTTAATTTTCG			
*Apidaecin*	*APID*	XM_006572699	F: GGCACGAGAAGAATTTTGCCT	59	Toll	This study
			R: GAAGGCGCGTAGGTCGAGTA			
*Defensin 1*	*Def1*	NM_001011616	F: TGCGCTGCTAACTGTCTCAG	59	Toll	This study
			R: AATGGCACTTAACCGAAACG			
*Protein groucho-1*	*PG1*	XM_006565410	F: ACAAGGATATAGGCCATAGCGAC	59	Wnt	This study
			R: GGTGGCGGGACTTCTTTCTT			
*Armadillo segment polarity protein*	*ARM*	XM_006557800	F: ATTCGAGGCAAGAAAGGCC	59	Wnt	This study
			R: ATCTACCCACACCGTATCGC			
*cAMP-dependent protein kinase catalytic subunit PRKX*	*PRKX*	XM_393711	F: ATATAGTCGAGCGACGAGCG	59	Wnt	This study
			R: CGAAAGTGCCTGTACCTATCGT			
*RING-box protein 1A*	*RBP*	XM_016910739	F: GTGGGAGTTCCAAAAATACGGT	59	Wnt	This study
			R: GAAGTGCTGCAAAGACGCTC			
*Protein phosphatase 2A at 29B*	*PP2A*	XM_006562757	F: AGGGTAATGCTTCCAACAGTCT	59	Wnt	This study
			R: CTTGTTCAACCTGCGATGCC			
*Protein phosphatase 2B at 14D*	*PP2B*	XM_016911698	F: TCCTCCTGTTTGTGTGGCAG	59	Wnt	This study
			R: GCTTTCCTCTCTCAGGACGG			
*Mothers against decapentaplegic homolog 4*	*SMAD4*	XM_006563458	F: CACACCGTAGGTAGCCAACA	59	Wnt	This study
			R:GCAGTACCTACTAAAGATGCTGCT			
*Ras-like GTP-binding protein Rho1*	*Rho1*	XM_016911392	F: GCGTGTGAGTGTCAAGCTGT	59	Wnt	This study
			R: CCTTCAAATCCAGCTCTTGC			

Organization of primers used in this study by gene name, abbreviation (Abbr.), accession no. (NCBI), Primer sequence (forward, F; reverse, R), melting temperature (T_m_), relevant pathways, and references.
